# Abnormalities of the *FHIT* gene in human oral carcinogenesis

**DOI:** 10.1054/bjoc.1999.1009

**Published:** 2000-02

**Authors:** K Tanimoto, S Hayashi, E Tsuchiya, Y Tokuchi, Y Kobayashi, K Yoshiga, T Okui, M Kobayashi, T Ichikawa

**Affiliations:** Department of Oral and Maxillofacial Surgery I, Hiroshima University School of Dentistry, 1-2- Kasumi, Minami-ku, Hiroshima, 734-8553, Japan; Departments of Biochemistry and Pathology, Saitama Cancer Center Research Institute, 818 Komuro, Ina-machi, Kitaadachi-gun, Saitama, 362-0806, Japan

**Keywords:** FHIT, gene alteration, microdissection, oral SCC, oral leukoplakia

## Abstract

The abnormalities of the fragile histidine triad (*FHIT*) gene in tissue samples of oral squamous cell carcinomas (SCCs) along with several leukoplakias and an erythroplakia were examined to determine whether the *FHIT* gene is actually a frequent target in vivo for alteration during oral carcinogenesis. Abnormal transcripts of the *FHIT* gene were found in eight of 15 oral SCCs. Although these abnormal transcripts varied widely, deletion patterns incorporating a deletion of exon 5 were the most common. Loss of heterozygosity (LOH) analysis demonstrated that the abnormal FHIT transcripts found in cancer cells were attributable to abnormalities of the *FHIT* gene. Abnormal FHIT transcripts were also observed in two of seven premalignant lesions. Interestingly, in the case of one patient with a premalignant lesion showing an abnormal FHIT transcript, subsequent oral SCC developed during a 3-year follow-up period. On the other hand, in the two patients from whom both leukoplakia and SCC samples were taken simultaneously, abnormal FHIT transcripts were found only in the SCCs. Although the functional role of FHIT remains to be clarified, these results suggest that the FHIT alteration is actually involved in carcinogenesis of the oral epithelium. © 2000 Cancer Research Campaign


*British Journal of Cancer* (2000) **82**(4), 838–843
© 2000 Cancer Research Campaign
DOI: 10.1054/ bjoc.1999.1009, available online at http://www.idealibrary.com on IDEAL®

### Abnormalities of the *FHIT* gene in human oral carcinogenesis

**K Tanimoto[1], S Hayashi[2], E Tsuchiya[3], Y Tokuchi[3], Y Kobayashi[3], K Yoshiga[1], T Okui[1], M Kobayashi[1] and T Ichikawa[1]**

[1]Department of Oral and Maxillofacial Surgery I, Hiroshima University School of Dentistry, 1-2- Kasumi, Minami-ku, Hiroshima 734-8553, Japan; Departments of [2]Biochemistry and [3]Pathology, Saitama Cancer Center Research Institute, 818 Komuro, Ina-machi, Kitaadachi-gun, Saitama 362-0806, Japan

**Summary** The abnormalities of the fragile histidine triad (*FHIT*) gene in tissue samples of oral squamous cell carcinomas (SCCs) along with several leukoplakias and an erythroplakia were examined to determine whether the *FHIT* gene is actually a frequent target in vivo for alteration during oral carcinogenesis. Abnormal transcripts of the *FHIT* gene were found in eight of 15 oral SCCs. Although these abnormal transcripts varied widely, deletion patterns incorporating a deletion of exon 5 were the most common. Loss of heterozygosity (LOH) analysis demonstrated that the abnormal FHIT transcripts found in cancer cells were attributable to abnormalities of the *FHIT* gene. Abnormal FHIT transcripts were also observed in two of seven premalignant lesions. Interestingly, in the case of one patient with a premalignant lesion showing an abnormal FHIT transcript, subsequent oral SCC developed during a 3-year follow-up period. On the other hand, in the two patients from whom both leukoplakia and SCC samples were taken simultaneously, abnormal FHIT transcripts were found only in the SCCs. Although the functional role of FHIT remains to be clarified, these results suggest that the FHIT alteration is actually involved in carcinogenesis of the oral epithelium. © 2000 Cancer Research Campaign

**Keywords:** FHIT; gene alteration; microdissection; oral SCC; oral leukoplakia

Oral cancer is one of the ten most common malignancies in the world, with a prevalence varying from 1–2% of total malignancies in Japan, the UK and the USA to 30–40% in India, Bangladesh and Pakistan, and with 90–95% of all cases presenting histologically as squamous cell carcinomas (SCCs) (Johnson, 1991). The pathogenesis of tumour development in the oral cavity is still not well understood, but oral leukoplakia and erythroplakia are widely recognized as the major premalignant states of oral SCCs. The lesions, which are defined clinically as white and red patches, initially show benign hyperkeratosis, and then, in more than 10% of the lesions showing microscopic dysplasia, transform to SCCs within a decade (Silverman et al, 1984).

It is well accepted that multiple genetic alterations are involved in the tumorigenic process of human cancers, and both mutation or overexpression of oncogenes and loss of tumour suppressor genes are often observed in various cancer tissues and cell lines. Complex patterns of such alterations have also been reported during the carcinogenesis of oral SCCs, and the tumour is believed to progress through a series of accumulations of genetic alterations in the same manner as other cancers (Papadimitrakopoulou et al, 1996; Todd et al, 1997).

Recently, the human fragile histidine triad (*FHIT*) gene has been identified at 3p14.2 using an exon-trapping strategy from cosmids covering this region, including the FRA3B fragile site in an epithelial cancer cell line (Ohta et al, 1996). It has been extensively reported that aberrantly spliced FHIT transcripts are frequently expressed in digestive tract, lung and breast cancers (Ohta et al, 1996; Sozzi et al, 1996; Hayashi et al, 1997). The *FHIT* gene alterations in head and neck cancers, including oral SCCs, have also been examined using SCC-derived cell lines (Mao et al, 1996; Virgilio et al, 1996), because, among various genetic alterations, loss of heterozygosity (LOH) at 3 p has frequently been reported in oral SCCs (Maestro et al, 1993; Ah-See et al, 1994). However, the importance of FHIT abnormalities during the process of oral cancer development in vivo, and particularly their possible association with hyperplastic or dysplastic lesions, is still unclear. In this study, we examined *FHIT* gene abnormalities in oral SCCs to determine whether the *FHIT* gene is actually a frequent target for alteration during oral tumorigenesis in vivo. To estimate the stage of involvement of the *FHIT* gene alterations, tissue samples of several leukoplakias and an erythroplakia were also subjected to study.

### MATERIALS AND METHODS

### Tissue samples

Twenty-two tissue specimens, including 15 primary oral SCCs, six leukoplakias and one erythroplakia, were obtained from 20 patients, and these patients, each of whom was subsequently monitored for at least 3 years at our clinic. The tumours were staged according to the TNM classification of malignant tumours defined by UICC (1987), and the patients were interviewed by the authors in regard to their smoking and drinking habits using a standardized questionnaire. The clinicopathological characteristics of these cases are summarized in Table 1. In two patients, a leukoplakia specimen and an oral SCC specimen were excised simultaneously: in patient No. 7 the leukoplakia specimen was from the upper gingiva on the opposite side of the incipient SCC; in patient No. 15 it was from a site adjacent to the buccal SCC. All specimens were

*Received 3 September 1998*
*Revised 19 August 1999*
*Accepted 26 August 1999*

*Correspondence to:* K Tanimoto: Department of Cell and Molecular Biology, Medical Nobel Institute, Karolinska Institutet, S-171 77 Stockholm, Sweden

**Table 1** Clinicopathological characteristics of oral SCC, leukoplakia and erythroplakia patients

| No. | Age | Sex | Subsite | Clinical diagnosis | Pathological diagnosis | TNM | Smoking/Drinking |
|---|---|---|---|---|---|---|---|
| 1 | 71 | F | Lower gingiva | Oral cancer | SCC | T2N0M0 | –/– |
| 2 | 64 | F | Tongue | Oral cancer | SCC | T4N2cM0 | –/– |
| 3 | 85 | F | Floor of mouth | Oral cancer | SCC | T4N2cM0 | –/– |
| 4 | 74 | M | Floor of mouth | Oral cancer | SCC | T4N3M1 | +/+ |
| 5 | 64 | F | Tongue | Oral cancer | SCC | T4N1M0 | –/– |
| 6 | 67 | F | Upper gingiva | Oral cancer | SCC | T4N0M0 | –/– |
| 7 | 56 | F | Upper gingiva (left side) | Oral cancer | SCC | T1N0M0 | –/+ |
| | | | [Upper gingiva (right side) | Leukoplakia | Dysplasia (mild) | – | |
| 8 | 74 | M | Upper gingiva | Oral cancer | SCC | T4N0M0 | –/– |
| 9 | 66 | F | Tongue | Oral cancer | SCC | T3N0M0 | –/– |
| 10 | 81 | F | Buccal mucosa | Oral cancer | SCC | T2N1M0 | –/– |
| 11 | 51 | M | Tongue | Oral cancer | SCC | T3N0M0 | +/+ |
| 12 | 69 | M | Lower gingiva | Oral cancer | SCC | T4N1M0 | +/+ |
| 13 | 78 | F | Tongue | Oral cancer | SCC | T2NxMx | –/– |
| 14 | 82 | F | Lower gingiva | Oral cancer | SCC | T3N1M0 | –/– |
| 15 | 79 | M | Buccal mucosa | Oral cancer | SCC | T1N0Mx | –/– |
| | | | [Buccal mucosa (adjacent site) | Leukoplakia | Dysplasia (moderate) | – | |
| 16 | 51 | M | Lower gingiva | Leukoplakia | Hyperplasia | – | +/+ |
| 17 | 53 | M | Lower gingiva | Leukoplakia | Hyperplasia | – | +/– |
| 18 | 69 | F | Upper gingiva | Erythroplakia | Dysplasia (moderate) | – | –/– |
| 19 | 59 | F | Tongue | Leukoplakia | Dysplasia (mild) | – | –/– |
| 20 | 59 | F | Tongue | Leukoplakia | Dysplasia (mild) | – | –/– |

M, male; F, female; SCC, squamouns cell carcinoma.

excised prior to any other treatment, such as radiation or chemotherapy, and cut into two pieces. One half of each sample was subjected to isolation of RNAs immediately; the other portion, including the surrounding normal tissue, was fixed in 10% formalin solution for routine microscopic examination. Specimens of apparently normal oral epithelia were also obtained from nine cancer-free individuals as controls.

### Preparation of RNA and RT-PCR

Total RNAs were prepared from 10–100 mg of human oral tissue following the method of Chomczynski and Sacchi (1987). Reverse transcription-polymerase chain reaction (RT-PCR) was carried out as described previously (Hayashi et al, 1997). Oligonucleotides used in PCR amplification of FHIT transcripts were as follows: P1 (sense strand in exons 1 and 2 of the *FHIT* gene according to Ohta et al (1996)), ATCCTGGAAGCTTTGAAGCTCA; P2 (anti-sense strand in exon 10), TCACTGGTTGAAGAATACAGGA; P3 (sense strand in exon 3), TCCGTAGTGCTATCTACATCC; P4 (anti-sense strand in exon 10), CATGCTGATTCAGTTCCTCTTG. The prepared RNAs (1 μg each) were reverse-transcribed to synthesize cDNA using random hexamers at 42°C and then subjected to the first PCR amplification with primers P1 and P2 in 20 μl of a mixture containing 10 mM Tris–HCl (pH 8.3), 50 mM potassium chloride (KCl), 1.5 mM magnesium chloride ($MgCl_2$) 0.01% gelatin and 0.2 mM dNTPs (dATP, dTTP, dGTP and dCTP). PCR was performed using a GeneAmp™ PCR System 9600 (Perkin-Elmer Cetus, Norwalk, CT, USA) and consisted of 25 cycles of denaturing at 95°C for 30 s, annealing at 65°C for 30 s and extension at 72°C for 1 min. The amplified mixtures were diluted 20-fold in 10 mM Tris–HCl (pH 7.5) buffer containing 1 mM EDTA, and 1 μl of the diluted mixtures was subjected to the second round of PCR using the nested primers P3 and P4 for 30 cycles under the above conditions. The PCR products were then subjected to 2% agarose gel electrophoresis and visualized by ethidium bromide staining. The fractionated DNAs were transferred to a nylon membrane (Hybond-N; Amarsham, Arlington Heights, IL, USA) and cross-linked under ultraviolet light. The oligonucleotide encoding exon 10 was labelled with [$\alpha$-$^{32}$P]dATP using the random primer method, and then used as a probe for hybridization in 5 × SSC (saline-sodium citrate), 10 × Denhardt's solution, 10 mM EDTA, 200 mg/ml of salmon sperm DNA and 1% sodium dodecyl sulphate (SDS) at 65°C. Radioactivity was then evaluated by autoradiography with the Fuji Bio-Image Analyser BAS 2000 (Fuji Film Co. Ltd, Tokyo, Japan).

### cDNA sequencing

PCR products were cut from gels. DNAs were purified using a Geneclean III Kit (BIO 101, Inc., Vista, CA, USA) and then subcloned in plasmid vector pGEM7Zf(+) (Promega, Madison, WI, USA). Sequencing was carried out using an AutoRead Sequencing Kit (Pharmacia Biotech, Uppsala, Sweden) and an A.L.F. DNA Sequencer II (Pharmacia LKB Biotechnology AB, Uppsala, Sweden) with fluoro-labelled SP6 and T7 primers.

### Microdissection and preparation of DNA

Genomic DNAs of six oral SCCs that had expressed abnormal FHIT transcripts were prepared from the formalin-fixed, paraffin-embedded tissue specimens using a microdissection technique (Gupta et al, 1997). Briefly, the regions of SCC and normal epithe-

*© 2000 Cancer Research Campaign* 

lium were identified and precisely dissected from the tissue sections under microscopic visualization. The scraped cells were subsequently pelleted by centrifugation, and DNAs were extracted by digesting the cells with a buffer containing 10 mM Tris–HCl (pH 7.5), 50 mM KCl, 1 mM EDTA, 1% SDS and 200 mg/ml proteinase K at 37°C for 24–36 h, followed by 10 min of incubation at 100°C to destroy any remaining proteinase K activity. The insoluble materials were pelleted by centrifugation, and aliquots of supernatant were used directly in PCR reactions.

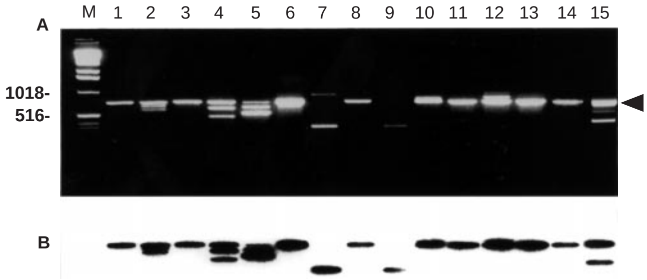

**Figure 1** Abnormalities of the FHIT transcripts in oral SCC. **(A)** Total RNAs prepared from SCC tissues were analysed by nested RT-PCR as described in Materials and Methods. A normal-size PCR product is indicated by an arrow head. Patient numbers shown at the top of each lane are matched with those in Table 1. Markers: commercial molecular weight marker (DNA ladder, BRL). **(B)** Southern blot hybridization of RT-PCR products was performed using a labelled oligo-probe encoding within exon 10 of the *FHIT* gene

### LOH analysis

Analysis of allelic losses was performed using a PCR-based approach (Tsuchiya et al, 1992; Hung et al, 1995). Primers that amplified polymorphic microsatellite markers were used for the locus, D3S1300, which was at intron 5 of the *FHIT* gene. After initial denaturation at 94°C for 4 min, 25 cycles of PCR were performed, each consisting of 30 s at 94°C, 30 s at 65°C and 30 s at 72°C. This was followed by 15 cycles consisting of 30 s at 90°C, 30 s at 65°C and 30 s at 72°C for denaturation, annealing and extension, respectively, with the final extension at 72°C for 2 min. PCR products were separated on a 6% urea-polyacrylamide gel, and then radioactivity was evaluated by autoradiography with a Fuji Bio-Image Analyser BAS 2000. For informative cases, allelic loss was scored if the autoradiographic signal of one allele was reduced in the tumor DNA, compared with the corresponding normal allele.

### RESULTS AND DISCUSSION

### Abnormal FHIT transcripts in human oral SCCs

Total RNAs were prepared from 15 oral SCC tissue specimens. RT-PCR analysis of SCC samples exhibited abnormalities of the FHIT transcription in eight of 15 cases (53%) (Figure 1A). However, none of nine normal oral epitheliums showed such abnormalities (data not shown). Southern blot hybridization, using an oligo-probe encoding within exon 10, verified that these products were derived from the *FHIT* gene (Figure 1B). Most of the

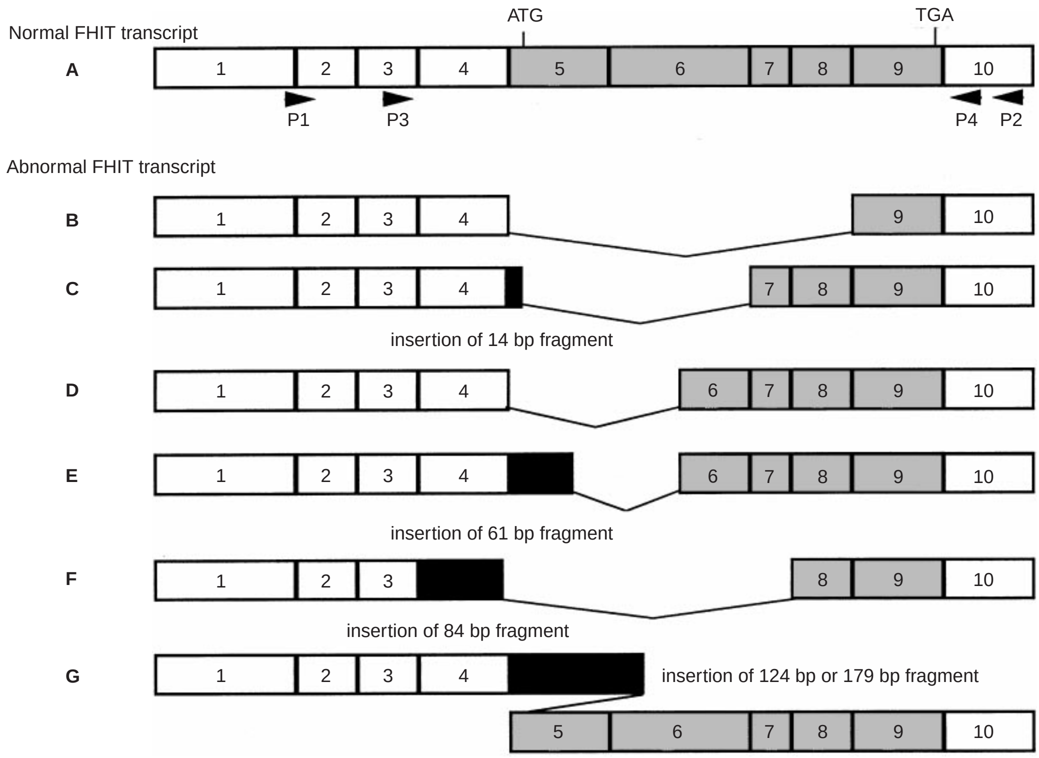

**Figure 2** Sequence analysis of various patterns of the FHIT transcripts in oral SCC. White, shaded and closed boxes indicate untranslated exons, coding exons and insertions, respectively. Solid lines linking these exons indicate the skipped regions. Arrow heads indicate the primers for RT-PCR. **(A)** Normal FHIT transcript, **(B)** deletion of exons 5–8, **(C)** deletion of exons 5 and 6, insertion of 14 bp fragment, **(D)** deletion of exon 5, partial deletion of exon 6, **(E)** deletion of exon 5, partial deletion of exon 6, insertion of 61 bp fragment, **(F)** deletion of exons 4–7, insertion of 84 bp fragment, **(G)** insertion of 124 bp or 179 bp fragment

 *© 2000 Cancer Research Campaign*

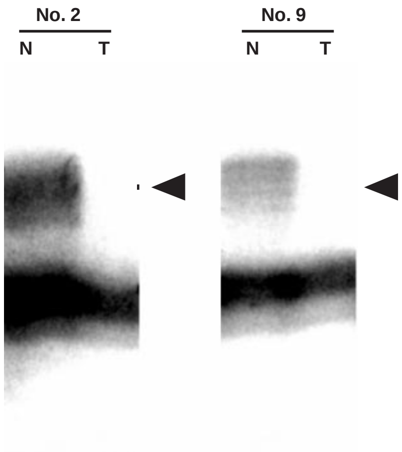

**Figure 3** Representative examples of LOH at the 3p14.2 locus in oral SCC. LOH analyses were performed using a microsatellite marker, D3S1300, as described in Materials and Methods, and two LOH cases (Nos 2 and 9) are depicted. N: paired normal tissue; T: tumour (SCC)

**Table 2** Abnormalities of FHIT in oral SCC patients

| Patient No | FHIT transcript | *FHIT* gene |
|---|---|---|
| 1 | Normal | – |
| 2 | Deletion of exon 5; partial deletion of exon 6; insertion of 61 bp fragment | LOH |
| 3 | Normal | – |
| 4 | Deletion of exon 5 and 6; insertion of 14 bp fragment | NI |
| 5 | Deletion of exon 5; partial deletion of exon 6 | NI |
| 6 | Normal | – |
| 7 | Deletion of exon 5 to 8 | LOH |
| 8 | Normal | – |
| 9 | Deletion of exon 5 to 8 | LOH |
| 10 | Insertion of 124 bp fragment | – |
| 11 | Normal | – |
| 12 | Insertion of 179 bp fragment | – |
| 13 | Normal | – |
| 14 | Normal | – |
| 15 | Deletion of exon 4 to 7; insertion of 84 bp fragment | LOH |

NI, not informative; –, not detected.

cases containing abnormal transcripts displayed both normal- and abnormal-sized RT-PCR products, with abnormal-sized products observed only in case Nos 7 and 9. Our results indicated that abnormal FHIT transcripts occurred frequently in oral SCCs in vivo, and thus agreed with the observations of previous in vitro works using SCCs-derived cell lines (Mao et al, 1996; Virgilio et al, 1996). Sequence analysis of RT-PCR products showed that the deletion of exons 5 through 6, or through 8, of the FHIT cDNA, which included the starting ATG codon of the FHIT protein, was the most common abnormality. In these cases, various lengths (14 bp to 179 bp) of fragments which were unrelated to the sequence of the *FHIT* gene were also observed (Figure 2). Extra bands having a slightly larger size than that of the normal FHIT transcript were found in two cases (Nos 10 and 12). Sequence analysis confirmed that these bands represented FHIT transcripts with a 124-bp or 179-bp insertion of unrelated fragments between exons 4 and 5 respectively (data not shown). These insertions, which seemed to be similar to those previously reported (Virgilio et al, 1996; Fong et al, 1997), may affect translation fidelity, but the role of such larger-sized transcripts is still unclear.

The simultaneous presence of wild-type and abnormal transcripts observed here has also been seen in many other kinds of tumour tissues and cell lines. It has been suggested that partial deletions of the *FHIT* gene might affect transcription fidelity and result in varying levels of abnormal transcripts. Mao suggested another possibility, that abnormal transcripts might be derived from subclones, since the *FHIT* gene contains a fragile site, FRA3B (1998). Such mutations, though often seen in other tumour suppressor genes, have not often been observed in the FHIT alleles in tumour tissues and cell lines, and thus these findings may contradict the notion that the *FHIT* gene is a classic tumour suppressor gene in terms of its inactivation patterns and functions. These results raise the possibility that the abnormal transcripts may inhibit protein translations or trigger protein degradations, or that they may encode truncated proteins which could act in a dominant-negative manner.

### LOH analysis of the *FHIT* gene with microdissected DNA

Because the abnormal FHIT transcripts observed in various tissues and cell lines, including non-neoplastic cells (Panagopoulos et al, 1997), might have included the products of alternative splicing, we performed LOH analysis using the microsatellite marker D3S1300 located near exon 5 to confirm that these abnormalities could actually be attributed to the *FHIT* gene. To eliminate possible contamination of non-neoplastic cells, microdissection from formalin-fixed sections of paraffin-embedded tumour samples was performed for preparation of genomic DNAs from oral SCC cells for LOH analysis. The results showed that allelic loss was present in four of six SCC specimens that expressed abnormal FHIT transcripts, with the other two cases being not informative (examples in Figure 3 and Table 2). These findings suggest that the abnormal FHIT transcripts found in this study can be attributed to abnormalities of the *FHIT* gene, not to alternatively spliced transcripts.

A recent study found that LOH at the locus of the *FHIT* gene was significantly more common in a group of smokers with lung cancer than in a group of non-smokers with lung cancer (Sozzi et al, 1997). Because epidemiological data have indicated a significant association between cigarette smoking and development of oral cancer (Takezaki et al, 1996), we compared the clinicopathological characteristics of oral SCC patients with the status of FHIT abnormalities (Table 1 and 2). However, FHIT status was not correlated with any epidemiological factor, including age at cancer onset, sex, subsite, TNM classification, or tobacco or alcohol consumption. The oral cavity is continuously exposed to numerous compounds, including possible carcinogens, and the pathway of oral tumorigenesis is considered to be more complicated than that of the lung (Tanimoto et al, 1999), which may be one reason that we were unable to discover any intimate relationship between clinical characteristics and FHIT status in oral SCCs.

### FHIT transcripts in human oral leukoplakia and erythroplakia

FHIT transcripts from seven patients, six with leukoplakias and one with erythroplakia, which included both histological epithelial

© 2000 Cancer Research Campaign

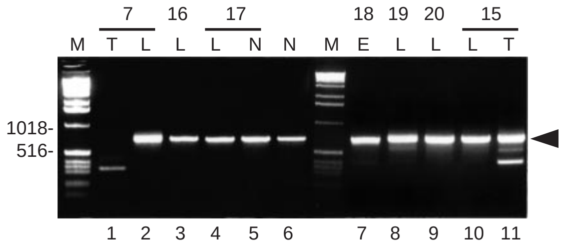

**Figure 4** Abnormalities of the FHIT transcripts in leukoplakias and an erythroplakia. Total RNAs prepared from leukoplakias and an erythroplakia were analysed by nested RT-PCR as described in Materials and Methods. Each PCR reaction was performed twice and confirmed by Southern blot hybridization and sequencing (data not shown). A normal-size PCR product is indicated by an arrow head. M: molecular weight marker, T: tumour (SCC); L: leukoplakia; E: erythroplakia; N: normal epithelium hyperplasia and from slight to moderate dysplasia, were examined to evaluate the involvement of FHIT abnormalities in the premalignant lesions. Abnormal FHIT transcripts were found in two of these samples (Nos 18 and 19; Figure 4). All patients were monitored for at least 3 years after the excisions; with the exception of patient No. 18 – in whom oral SCC arose at the upper gingival site of the excision of the erythroplakia with abnormal FHIT transcripts – no signs of malignant change were observed in any of the patients. Comparing SCC and leukoplakia samples, both of which were obtained simultaneously from the oral SCC patients, abnormal FHIT transcripts were observed in tumors but not in leukoplakias (Nos 7 and 15; Figure 4).

The occurrence of multiple oral cancers and the high incidence of the second primary oral cancers can be explained by the field cancerization theory, which proposes that prolonged exposure to carcinogens leads to the independent transformation of multiple epithelial cells over the entire exposed field (Slaughter et al, 1953; Liciardello et al, 1989). This theory suggests that leukoplakias that arise together with malignant lesions, as in two of our patients, might have already accumulated genetic damages; to investigate this possibility, we compared our simultaneusly obtained leukoplakia and SCC samples. In both cases, however, abnormal FHIT transcripts were observed only in tumours, not in leukoplakia samples (Nos 7 and 15; Figure 4). The *FHIT* gene, which is similar to a yeast diadenosine hydrolase gene (Barnes et al, 1996), was expected to be a candidate as a putative tumour suppressor gene, but it has recently been suggested that the *FHIT* gene is not a selective target and that the 3p14 deletion results from the genomic instability that may correlate in part with *p53* gene inactivation (Boldog et al, 1997). Recently, Siprashvili et al (1997) showed that transfection of the *FHIT* gene into tumour cell lines which lacked endogenous FHIT protein could significantly reduce tumour formation and tumour size in nude mice but not in vitro. Furthermore, their experiments using FHIT mutants indicated that diadenosine hydrolase was not required for tumour suppression. In contrast, Otterson et al (1998) showed that expression of the FHIT cDNA construct did not change the cell proliferation or alter tumorigenicity in animals. Interestingly, in both studies, researchers observed no consistent effect of exogenous FHIT expression on cell growth. In this study, it was elucidated that the alteration of the *FHIT* gene is frequently seen in oral SCCs. However, as mentioned above, the functional role of the *FHIT* gene in the process of carcinogenesis is not yet well understood, and, at present, the possibility of its being a tumour suppressor gene is rather controversial.

Our study scale, particularly with respect to the premalignant lesions, was too small to reach definite conclusions; however, our findings suggest that FHIT abnormalities may not simply reflect accumulation of genetic damage, and may work as a sensor of genetic alterations rather than as a classical tumour suppressor during oral carcinogenesis. If so, analysis of *FHIT* gene abnormalities in small biopsy specimens may help to identify premalignant lesions that already possess the potential to transform to malignancies. To confirm this possibility, further investigation of the FHIT alterations in various grades of epithelial dysplasia will be needed.

### REFERENCES

Ah-See KW, Cooke TG, Pickford IR, Soutar D and Balmain A (1994) An allelotype of squamous carcinoma of the head and neck using microsatellite markers. *Cancer Res* **54:** 1617–1621

Barnes LD, Garrison PN, Siprashvili Z, Guranowski A, Robinson AK, Ingram SW, Croce CM, Ohta M and Huebner K (1996) Fhit, a putative tumor suppressor in humans, is a dinucleoside 5′, 5‴-P1,P3-triphosphate hydrolase. *Biochemistry* **35:** 11529–11535

Boldog F, Gemmill RM, West J, Robinson M, Robinson L, Li E, Roche J, Todd S, Waggoner B, Lundstrom R, Jacobson J, Mullokandov MR, Klinger H and Drabkin HA (1997) Chromosome 3p14 homozygous deletions and sequence analysis of FRA3B. *Hum Mol Genet* **6:** 193–203

Chomczynski P and Sacchi N (1987) Single-step method of RNA isolation by acid guanidinium thiocyanate–phenol–chloroform extraction. *Anal Biochem* **162:** 156–159

Fong KM, Biesterveld EJ, Virmani A, Wistuba I, Sekido Y, Bader SA, Ahmadian M, Ong ST, Rassool FV, Zimmerman PV, Giaccone G, Gazdar AF and Minna JD (1997) FHIT and FRA3B 3p14.2 allele loss are common in lung cancer and preneoplastic bronchial lesions and associated with cancer-related FHIT cDNA splicing aberrations. *Cancer Res* **57:** 2256–2267

Gupta SK, Douglas-Jones AG and Morgan JM (1997) Microdissection of stained archival tissue. *Mol Pathol* **50:** 218–220

Hayashi SI, Tanimoto K, Hajiro-Nakanishi K, Tsuchiya E, Kurosumi M, Higashi Y, Imai K, Suga K and Nakachi K (1997) Abnormal FHIT transcripts in human breast carcinomas: a clinicopathological and epidemiological analysis of 61 Japanese cases. *Cancer Res* **57:** 1981–1985

Hung J, Kishimoto Y, Sugio K, Virmani A, Mclntire DD, Minna JD and Gazdar AF (1995) Allele-specific chromosome 3p deletions occur at an early stage in the pathogenesis of lung carcinoma. *JAMA* **273:** 1908

Johnson NW (1991) A global view of the epidemiology of oral cancer. In: *Oral Cancer: The Detection of Patients and Lesions at Risk*, Johnson NW (ed), pp. 3–26. Cambridge University Press: Cambridge

Licciardello JTW, Spitz MR and Hong WK (1989) Multiple primary cancer in patients with cancer of the head and neck: second cancer of the head and neck, esophagus and lung. *Int J Radiat Oncol Biol Phys* **17:** 467–476

Maestro R, Gasparotto D, Vukosavljevic T, Barzan L, Sulfaro S and Boiocchi M (1993) Three discrete regions of deletion at 3p in head and neck cancers. *Cancer Res* **53:** 5775–5779

Mao L (1998) Tumor suppressor gene: does FHIT fit? *J Natl Cancer Inst* **90:** 412–414

Mao L, Fan YH, Lotan R and Hong WK (1996) Frequent abnormalities of FHIT, a candidate tumor suppressor gene, in head and neck cancer cell lines. *Cancer Res* **56:** 5128–5131

Ohta M, Inoue H, Cotticelli MG, Kastury K, Baffa R, Palazzo J, Siparashvili Z, Mori M, McCue P, Druck T, Croce CM and Huebner K (1996) The FHIT gene, spanning the chromosome 3p14.2 fragile site and renal carcinoma-associated t(3;8) breakpoint, is abnormal in digestive tract cancers. *Cell* **84:** 587–597

Otterson GA, Xiao GH, Geradts J, Jin F, Chen Wd, Niklinska W, Kaye FJ and Yeung RS (1998) Protein expression and functional analysis of the FHIT gene in human tumor cells. *Int Natl Cancer Inst* **90:** 426–432

Panagopoulos I, Thelin S, Mertens F, Mitelman F and Aman P (1997) Variable FHIT transcripts in non-neoplastic tissues. *Genes Chromosomes Cancer* **19:** 215–219

Papadimitrakopoulou VA, Shin DM and Hong WK (1996) Molecular and cellular biomarkers for field cancerization and multistep process in head and neck tumorigenesis. *Cancer Metastasis Rev* **15:** 53–76

© 2000 Cancer Research Campaign

Silverman S Jr, Gorsky M and Lozada F (1984) Oral leukoplakia and malignant transformation. A follow-up study of 257 patients. *Cancer* **53:** 563–568

Siprashvili Z, Sozzi G, Barnes LD, McCue P, Robinson AK, Eryomin V, Sard L, Tagliabue E, Greco A, Fusetti L, Schwartz G, Pierotti MA, Croce CM and Huebner K (1997) Replacement of Fhit in cancer cells suppresses tumorigenicity. *Proc Natl Acad Sci USA* **94:** 13771–13776

Slaughter DP, Southwick HW and Smejkal W (1953) 'Field cancerization' in oral stratified squamous epithelium: clinical implications of multicentric origin. *Cancer* **6:** 963–968

Sozzi G, Veronese ML, Negrini M, Baffa R, Cotticelli MG, Inoue H, Tornielli S, Pilotti S, De Gregorio L, Pastorino U, Pierotti MA, Ohta M, Huebner K and Croce CM (1996) The FHIT gene 3p14.2 is abnormal in lung cancer. *Cell* **85:** 17–26

Sozzi G, Sard L, De Gregorio L, Marchetti A, Musso K, Buttitta F, Tornielli S, Pellegrini S, Veronese ML, Manenti G, Incarbone M, Chella A, Angeletti CA, Pastorino U, Huebner K, Bevilaqua G, Pilotti S, Croce CM and Pierotti MA (1997) Association between cigarette smoking and FHIT gene alterations in lung cancer. *Cancer Res* **57:** 2121–2123

Takezaki T, Hirose K, Inoue M, Hamajima N, Kuroishi T, Nakamura S, Koshikawa T, Matsuura H and Tajima K (1996) Tobacco, alcohol and dietary factors associated with the risk of oral cancer among Japanese. *Jpn J Cancer Res* **87:** 555–562

Tanimoto K, Hayashi SI, Yoshiga K and Ichikawa T (1999) Polymorphisms of the CYP1A1 and GSTM1 gene involved in oral squamous cell carcinoma in association with a cigarette dose. *Oral Oncol* **35:** 191–196

Todd R, Donoff RB and Wong DTW (1997) The molecular biology of oral carcinogenesis: toward a tumor progression model. *J Oral Maxillofac Surg* **55:** 613–623

Tsuchiya E, Nakamura Y, Weng SY, Nakagawa K, Tsuchiya S, Sugano H and Kitagawa T (1992) Allelotype of non-small-cell lung carcinoma: comparison between loss of heterozygosity in squamous cell carcinoma and adenocarcinoma. *Cancer Res* **52:** 2478–2481

Virgilio L, Shuster M, Gollin SM, Veronese ML, Ohta M, Huebner K and Croce CM (1996) FHIT gene alterations in head and neck squamous cell carcinomas. *Proc Natl Acad Sci USA* **93:** 9770–9775

*© 2000 Cancer Research Campaign*
